# The Wheat Nucleoredoxin *TaNRX1-2D* Gene Ameliorates Salt Tolerance in Wheat (*Triticum aestivum* L.)

**DOI:** 10.3390/plants15010146

**Published:** 2026-01-04

**Authors:** Jianfei Zhou, Xiling Chang, Yaning Bu, Tianqi Song, Ling Kang, Yan Dong, Xinpeng Lei, Yuxin Wang, Xiaoxing Wang, Jiandong Ren, Jishan Xiang, Dongsheng Chen, Xiaoke Zhang

**Affiliations:** 1 College of Agronomy, Northwest A&F University, Yangling 712100, China; zhoujianfei@nwafu.edu.cn (J.Z.); aau13625519527@163.com (X.C.); buyaning@nwafu.edu.cn (Y.B.); songtq@nwafu.edu.cn (T.S.); 18846915712@163.com (X.L.); 15234519991@163.com (Y.W.); 2 Crop Research Institute, Ningxia Academy of Agriculture and Forestry Science, Yinchuan 750002, China; kangling1005@163.com (L.K.); 18195164363@163.com (Y.D.); 3 College of Biological Sciences and Technology, Yili Normal University, Yili 830500, China; wangxiaoxing_yl@126.com (X.W.); jiandong_ren2020@126.com (J.R.)

**Keywords:** wheat, *TaNRX1-2D*, TaERD15L-3B, TaCAT2-B, salt stress

## Abstract

Wheat is one of the most important crops contributing to global food and nutritional security. However, the gradual increase in soil salt content significantly impairs wheat growth and development, ultimately resulting in reduced yields. Therefore, enhancing the salt tolerance of wheat is of significant importance. Salt stress commonly induces oxidative stress in plants, and nucleoredoxin (NRX) has been shown to effectively maintain redox homeostasis under stress conditions. However, the functional role and molecular mechanism of the *NRX* gene in regulating salt tolerance in wheat remain to be elucidated. The results of this study demonstrated that *TaNRX1-2D* homologous overexpression (OE) lines exhibited significantly enhanced tolerance to salt stress. The survival rate and antioxidant enzyme activities (including superoxide dismutase and catalase) in the OE lines were higher than those in the wild type (WT). In contrast, the levels of superoxide anion (O_2_^−^), hydrogen peroxide (H_2_O_2_), and malondialdehyde (MDA) in the OE lines were markedly lower than those in the WT. Conversely, the RNA interference (RNAi) lines displayed opposing trends. The results of yeast one-hybrid (Y1H) and dual luciferase assays (D-LUC) demonstrated that the TaERD15L-3B transcription factor positively regulated the expression of the *TaNRX1-2D* gene by binding to the ABRERATCAL *cis*-acting element in the *TaNRX1-2D* promoter. Through luciferase complementation assay (LCA), bimolecular fluorescence complementation (BiFC) assay, and a “mutation capture strategy”, it was found that TaNRX1-2D (C54, 327S) interacted with TaCAT2-B, indicating that TaCAT2-B was the target protein of TaNRX1-2D. The results of data-independent acquisition (DIA) proteomics analysis indicated that TaNRX1-2D may mediate salt tolerance in wheat through the positive regulation of nsLTP protein abundance and the negative regulation of hexokinase protein abundance. In general, the TaERD15L-3B/TaNRX1-2D regulatory module played a crucial role in conferring salt tolerance in wheat. This study provided an important theoretical basis and identified a potential gene target for developing salt-tolerant wheat varieties through molecular breeding approaches.

## 1. Introduction

As one of the most significant staple crops globally, wheat provides a substantial source of calories, proteins, vitamins, and micronutrients, contributing to global food and nutritional security [1]. However, due to rising sea levels caused by global climate change, inappropriate irrigation practices utilizing groundwater with high salt concentrations, and the accelerated pace of urbanization, soil salinity has become a critical environmental challenge [2]. Salt stress can impair the absorption of water and essential nutrients in wheat, while also inducing oxidative, ionic, and osmotic stresses, ultimately leading to a significant decline in both the yield and quality of wheat [3].

To mitigate the escalating threat of salt stress, wheat has evolved diverse adaptive defense mechanisms. Wheat salt tolerance is a complex quantitative trait controlled by multiple genes associated with processes including redox reactions, ion transport, and the accumulation of osmoregulatory substances [4,5]. Elucidating the genetic basis of salt tolerance regulation in wheat and comprehensively analyzing the underlying molecular mechanisms can provide critical insights for breeding salt-tolerant wheat varieties and establishing corresponding salt-tolerant cultivation systems.

Under normal conditions, ROS function as signaling molecules, contributing to various aspects of plant growth and stress perception. However, adverse environmental conditions typically result in elevated ROS levels within plant cells [6,7]. Excessive accumulation of ROS can lead to the oxidation and modification of protein thiol groups in plants, which may alter their spatial conformation and ultimately result in decreased activity or even complete loss of function [8]. Thioredoxins (TRXs) are ubiquitously present and highly conserved across plant species. TRXs can reduce misformed disulfide bonds in their target proteins through their characteristic active site motif containing two cysteine (Cys) residues, “-Cys-X-X-Cys- (-CXXC-, X denotes any amino acid excluding Cys)”, thereby contributing to the maintenance of cellular redox homeostasis [9,10]. Nucleoredoxin (NRX) is a member of the thioredoxin superfamily and derives its name from its initial identification in murine cells and nuclear localization observed following heterologous expression in COS-7 cells [11]. NRX exhibits a higher molecular weight and possesses more active sites compared to typical TRX. Following infection of island cotton (*Gossypium barbadense*) by *Verticillium dahliae*, a significant accumulation of ROS was observed in the extracellular matrix. Concurrently, the abundance of the GbNRX1 protein significantly increased, indicating its involvement in the clearance of excessive ROS produced in the extracellular matrix. The silencing of the *GbNRX1* gene via the virus-induced gene silencing (VIGS) system led to a reduction in the resistance of island cotton to *Verticillium dahliae* [12]. Following heat stress, the CRISPR/Cas9-mediated *slnrx1* mutant of tomato exhibited a significant increase in malondialdehyde (MDA) content and hydrogen peroxide (H_2_O_2_) concentration compared to the wild type (WT). SlNRX1 alleviated oxidative stress in tomatoes under heat stress through upregulation of genes encoding antioxidant enzymes and heat shock proteins (HSPs) [13]. The ROS burst induced by *Pseudomonas syringae* infection in *Arabidopsis* leads to the oxidation and inactivation of catalase (CAT), which is retained in the cytoplasm. AtNRX1 was able to directly target and reduce oxidized CAT, thereby restoring its catalytic activity and enabling the continued removal of excess H_2_O_2_, ultimately contributing to the maintenance of ROS homeostasis in infected *Arabidopsis* cells [14]. These results indicate that the NRX protein plays a crucial role in protecting plants against oxidative stress.

TRX employs two cysteine residues located in its active site to facilitate the reduction in target proteins containing disulfide bonds via a two-step reaction mechanism. This thiol-disulfide exchange process can be essentially characterized as an instantaneous electron transfer process [9,15]. If one cysteine residue within the active site of TRX is substituted with another amino acid residue, TRX is capable of forming a stable complex with its target protein. The method for identifying TRX target proteins based on this theory is referred to as the “mutation capture strategy” [16]. Motohashi et al. [16] initially employed this strategy to identify target proteins of the m subtype of TRX in spinach chloroplasts, and subsequently demonstrated through a series of biochemical experiments that TRX m is capable of directly targeting and reducing the oxidized forms of 2-Cys Peroxiredoxins (Prx) and cyclophilin proteins. Kneeshaw et al. [14] employed this strategy to identify 74 target proteins of NRX1 in *Arabidopsis thaliana* infected with *Pseudomonas syringae*. Gene Ontology (GO) analysis indicated that these target proteins were predominantly involved in electron transport and energy metabolism pathways. Identifying the target proteins of TRX or NRX contributes to the analysis and elucidation of their functional roles and underlying molecular mechanisms in plants.

Our previous research demonstrated that following drought treatment, wheat lines overexpressing the *TaNRX1-2D* (TraesCS2D02G466800) gene exhibited significantly higher antioxidant enzyme activities compared to the wild type (WT), resulting in an enhanced capacity to scavenge ROS [17]. The performance of wheat lines with the *TaNRX1-2D* gene silenced exhibited an opposite trend compared to those overexpressing the gene [17]. However, the functional role and molecular mechanism of the *TaNRX1-2D* gene in regulating salt stress tolerance in wheat remain to be fully elucidated. Abscisic acid (ABA) is a crucial plant hormone involved in mediating plant responses to adverse environmental stresses. Drought and salt stress can both induce the accumulation of ABA, thereby triggering a range of adaptive physiological and biochemical responses in plants [18]. The expression activity analysis of the GUS gene under the control of the 5′ deletion promoter revealed that the 36-bp fragment (−193 bp to −157 bp) within the *TaNRX1-2D* gene promoter served as the key regulatory region responsive to ABA signaling [19]. The 36-bp fragment contains two *cis*-acting elements associated with ABA signaling, namely ABRERATCAL and ACGTATERD1; however, the transcription factors that potentially bind to these elements and regulate the expression of the *TaNRX1-2D* gene remain to be identified. In this study, we aimed to (1) identify and analyze the function of *TaNRX1-2D* gene in wheat under salt stress conditions using *TaNRX1-2D* homologous transgenic lines; (2) conduct yeast one-hybrid (Y1H) assay to identify transcription factors capable of binding to the aforementioned *cis*-acting elements on the promoter of *TaNRX1-2D*, and subsequently perform dual-luciferase reporter assays (D-LUC) to elucidate the regulatory mechanisms by which the identified transcription factors influence the expression of *TaNRX1-2D*; (3) analyze the interaction between TaNRX1-2D and TaCAT protein using luciferase complementation assay (LCA), bimolecular fluorescence complementation assay (BiFC), and the “mutation capture strategy”; and (4) investigate the potential molecular mechanisms underlying *TaNRX1-2D*-mediated salt tolerance in wheat through proteomic profiling of *TaNRX1-2D* homologous transgenic lines under both pre- and post-salt stress conditions. These findings may provide valuable insights into the molecular mechanisms underlying the regulation of salt stress tolerance in wheat by the *TaNRX1-2D* gene, and identify potential candidate genes for the development of salt stress-tolerant wheat varieties.

## 2. Results

### 2.1. Identification of Salt Tolerance in TaNRX1-2D Transgenic Wheat

**Figure 1:**
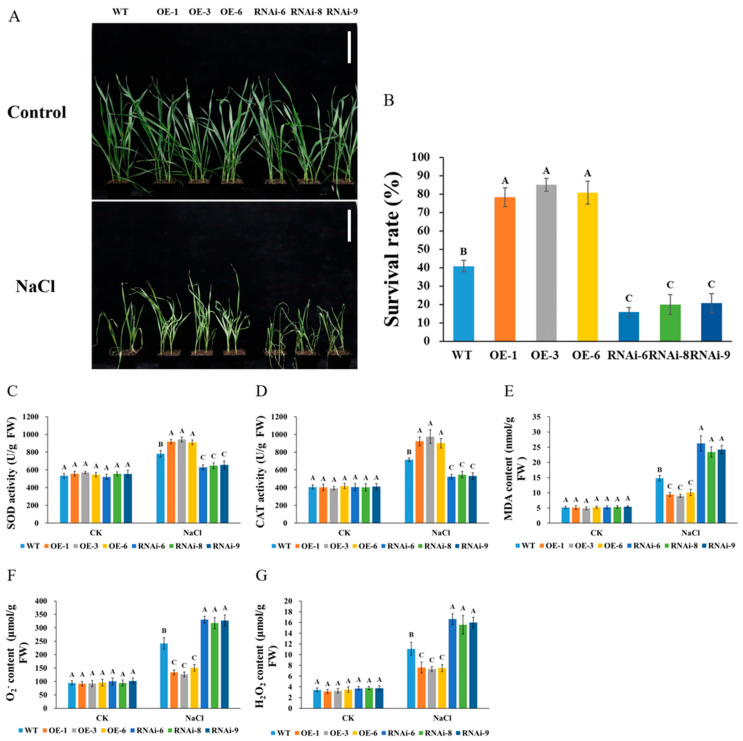
Phenotype (**A**), survival rate (**B**), and physiological indices (**C**–**G**) of WT and *TaNRX1-2D* transgenic wheat under normal (Control) and salt stress (200 mM NaCl) conditions at the seedling stage. (**C**) Enzyme activity of SOD. (**D**) Enzyme activity of CAT. (**E**) MDA content. (**F**) O_2_^−^ content. (**G**) H_2_O_2_ content. WT, wild type; OE-1–OE-6, *TaNRX1-2D* overexpression T_3_ homogeneous lines; RNAi-6–RNAi-9, *TaNRX1-2D* RNA interference T_3_ homogeneous lines. Vertical bars indicate standard deviations. Different capital letters indicate extremely significant differences at *p* < 0.01 within condition according to one-way ANOVA and post hoc Tukey’s test. Scale bars: 10 cm.

No significant phenotypic differences were observed between transgenic and WT lines under normal growth conditions during the seedling stage (Figure 1A). Salt stress induced more severe wilting in RNAi lines compared to the WT and OE lines (Figure 1A), resulting in a reduced survival rate (Figure 1B). In comparison with the WT and RNAi lines, the OE lines exhibited higher survival rates (Figure 1B). Following treatment with 200 mM NaCl, the antioxidant enzyme activities of SOD and CAT in the *TaNRX1-2D*-OE lines were significantly elevated compared to those in the WT plants. In contrast, the enzyme activities in the *TaNRX1-2D*-RNAi lines were markedly reduced relative to the WT (Figure 1C,D). Correspondingly, the levels of O_2_^−^, H_2_O_2_, and MDA in the OE lines were significantly lower than those in the WT line, whereas the levels of these three oxidative stress indicators in the RNAi lines were markedly elevated compared to those in the WT line (Figure 1E–G).

**Figure 2:**
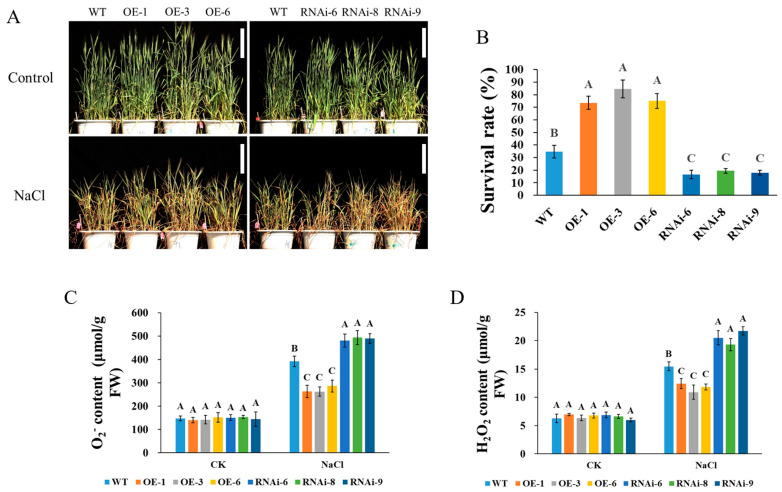
Phenotype (**A**), survival rate (**B**), O_2_^−^ content (**C**) and H_2_O_2_ content (**D**) of WT and *TaNRX1-2D* transgenic wheat under normal (Control) and salt stress (200 mM NaCl) conditions at the heading stage. To ensure consistency in comparison, we separately selected the same pot of WT wheat plants as controls in both the control and NaCl treatments (**A**). WT, wild type; OE-1–OE-6, *TaNRX1-2D* overexpression T_3_ homogeneous lines; RNAi-6–RNAi-9, *TaNRX1-2D* RNA interference T_3_ homogeneous lines. Vertical bars indicate standard deviations. Different capital letters indicate extremely significant differences at *p* < 0.01 within condition according to one-way ANOVA and post hoc Tukey’s test. Scale bars: 20 cm.

Similar to the observations during the seedling stage, when transgenic and WT lines were subjected to 200 mM NaCl treatment at the heading stage, the levels of O_2_^−^ and H_2_O_2_ in the RNAi lines were significantly higher than those in the WT lines (Figure 2C,D), leading to more pronounced symptoms of yellowing and wilting, as well as reduced survival rates in the RNAi lines (Figure 2A,B). In contrast, the OE lines exhibited significantly lower levels of O_2_^−^ and H_2_O_2_ compared to the WT, which corresponded with a higher survival rate (Figure 2B–D).

### 2.2. Screening and Identification of Transcription Factor Regulating TaNRX1-2D

#### 2.2.1. Screening of Transcription Factors Using Y1H Assay

The Y1HGold [pAbAi-(ABRE+ACGT)] yeast strain was almost unable to grow on SD/-Ura medium supplemented with 1000 ng/mL AbA, suggesting the absence of endogenous transcription factors in yeast capable of binding to either the ABRRATCAL or ACGTATERD1 elements under this AbA concentration. Therefore, a concentration of 1000 ng/mL AbA was suitable for screening yeast one-hybrid libraries.

After transferring the wheat leaf cDNA library plasmid into pAbAi-(ABRE+ACGT) yeast competent cells, the transformed cells were cultured on SD/-Ura/-Leu solid medium supplemented with 1000 ng/mL AbA for 3–5 d. Positive monoclonal colonies were selected and further amplified using universal primers (T7 and 3-AD) specific to the pGADT7 vector (Table S1). The resulting PCR products were analyzed by gel electrophoresis and subsequently sequenced. Sequence analysis was performed using the NCBI database, leading to the identification of a total of 21 proteins, including the transcription factors TaERD15L-3B (TraesCS3B02G409300) and TabHLH-6D (TraesCS6D02G038400) (Table S2).

The detection results of relative gene expression levels of *TaERD15L-3B* and *TabHLH-6D* under salt stress indicated that the expression level of *TaERD15L-3B* began to increase at 0.5 h after salt stress treatment and peaked at 6 h, reaching 5.1 times the level at 0 h (before salt stress treatment). Although the expression level of *TaERD15L-3B* decreased at 12 h and 24 h compared to 6 h, it remained higher than the level at 0 h. This suggested that *TaERD15L-3B* can be induced and upregulated by salt stress. In contrast, except for a slight increase in *TabHLH-6D* expression at 0.5 h, its expression levels at all other time points were significantly reduced compared to 0 h (Figure S2). Therefore, TaERD15L-3B was selected as a candidate transcription factor for further investigation.

**Figure 3:**
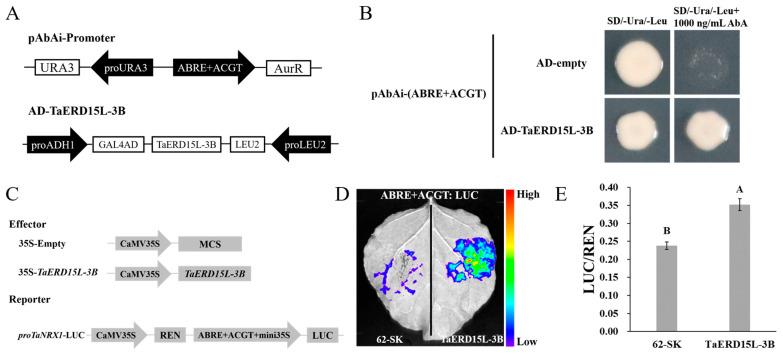
TaERD15L-3B binds to the promoter of *TaNRX1-2D* and regulates the expression of the *TaNRX1-2D* gene: (**A**) Schematic diagram of the structure of AD-*TaERD15L-3B* and pAbAi-*proTaNRX1-2D* carriers for yeast one-hybrid (Y1H). The *TaERD15L-3B* gene was inserted into the pGADT7 vector, and the *TaNRX1-2D* promoter was inserted into the pAbAi vector to form AD-*TaERD15L-3B* and pAbAi-*proTaNRX1-2D* recombinant vectors, respectively. (**B**) The yeast one-hybrid assay showed that the TaERD15L-3B transcription factor was directly bound to the *TaNRX1-2D* promoter. Among them, pAbAi-(ABRE+ACGT) and AD-empty were used as negative controls and cultured in SD/-Ura/-Leu plates containing 0 ng/mL AbA (as control) or 1000 ng/mL AbA, respectively. (**C**) Schematic diagram of effector and reporting factors for dual luciferase assay. (**D**,**E**) 35S: TaERD15L-3B and *proTaNRX1-2D*: LUC fluorescence imaging of LUC and quantitative analysis of fluorescence intensity in *Nicotiana benthamiana* leaves. Vertical bars indicate standard deviations. Different capital letters indicate extremely significant differences at *p* < 0.01 within condition according to one-way ANOVA and post hoc Tukey’s test.

The pGADT7-*TaERD15L-3B* recombinant vector plasmid and the pGADT7 empty vector plasmid were separately introduced into pAbAi-(ABRE+ACGT) yeast competent cells for Y1H validation (Figure 3A). The results demonstrated that yeast cells containing the pGADT7-*TaERD15L-3B* plasmid were able to grow normally on SD/-Ura/-Leu solid medium supplemented with 1000 ng/mL AbA, whereas cells transformed with the pGADT7 empty vector failed to grow under the same conditions (Figure 3B). These findings suggested that TaERD15L-3B was capable of interacting with the ABRERATCAL or ACGTATERD1 *cis*-acting element in yeast cells.

#### 2.2.2. Analysis of Regulatory Mechanisms Using D-LUC Assay

The dual-luciferase assay based on transient expression in tobacco can further elucidate the interaction and regulatory mechanisms between candidate transcription factors and the promoter sequences of target genes. The CDS sequence of *TaERD15L-3B* was inserted into the pGreen II-62-SK vector to generate the effector construct, and the “ABRE+ACGT” sequence, repeated thrice, was inserted into the pGreen II-0800-LUC vector to create the reporter construct (Figure 3C). The recombinant vector plasmid and the pGreen II-62-SK empty vector plasmid were separately introduced into GV3101 (pSoup-p19) *Agrobacterium* competent cells. To maintain a consistent experimental background, the left side of the tobacco leaf was infiltrated with the control combination consisting of pGreen II-62-SK and pGreen II-0800-LUC-(ABRE+ACGT), while the right side was infiltrated with the experimental combination containing pGreen II-62-SK-*TaERD15L-3B* and pGreen II-0800-LUC-(ABRE+ACGT). Following an adequate period of dark incubation, D-luciferin solution was uniformly applied as a reaction substrate to the leaf infiltrated sites, which were subsequently placed in the Plant View 100 imaging system to detect chemiluminescence signals. The results demonstrated that the regions of tobacco leaves co-infiltrated with pGreen II-62-SK-*TaERD15L-3B* and pGreenII-0800-LUC-(ABRE+ACGT) exhibited strong chemiluminescence signals, whereas the regions co-infiltrated with the control combination displayed significantly weaker signals (Figure 3D). Subsequently, equal amounts of samples were collected from the infiltrated sites on the tobacco leaves and were fully lysed to further assess the relative enzymatic activity of firefly luciferase (LUC) and renilla luciferase (REN). The results demonstrated that the LUC-to-REN ratio in tobacco leaf tissues co-infiltrated with the pGreen II-62-SK-*TaERD15L-3B* and pGreen II-0800-LUC-(ABRE+ACGT) constructs was significantly higher than that detected in the control combination (Figure 3E), suggesting that TaERD15L-3B could act as a positive regulatory factor of the *TaNRX1-2D* gene.

**Figure 4:**
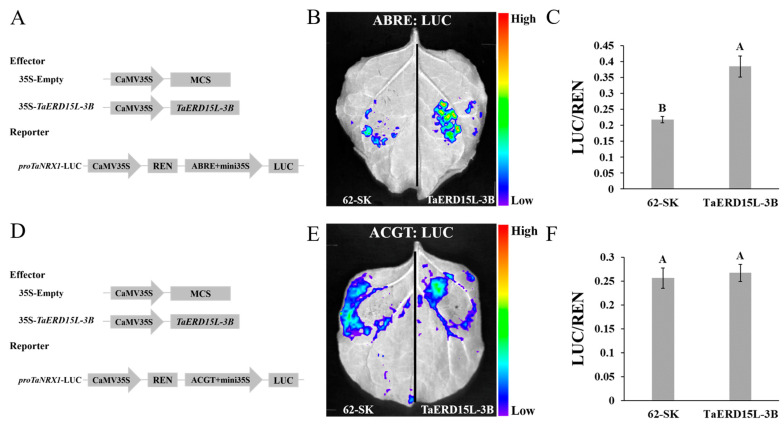
TaERD15L-3B binds to the ABRERATCAL *cis*-acting element of *TaNRX1-2D* promoter and regulates the expression of *TaNRX1-2D* gene: (**A**,**D**) Schematic diagram of effector and reporting factors for dual luciferase assay. (**B**,**E**) 35S: TaERD15L-3B and *proTaNRX1-2D*: LUC fluorescence imaging of LUC in *N. benthamiana* leaves. (**C**,**F**) 35S: TaERD15L-3B and *proTaNRX1-2D*: LUC quantitative analysis of fluorescence intensity in *N. benthamiana* leaves. Vertical bars indicate standard deviations. Different capital letters indicate extremely significant differences at *p* < 0.01 within condition according to one-way ANOVA and post hoc Tukey’s test.

To further investigate the *cis*-acting element specifically bound by TaERD15L-3B, two reporter recombinant vectors, pGreen II-0800-LUC-ABRE and pGreen II-0800-LUC-ACGT, were constructed (Figure 4A,D). Chemiluminescence imaging and luciferase activity assays revealed that the combination of pGreen II-62-SK-*TaERD15L-3B* with pGreen II-0800-LUC-ABRE exhibited a stronger chemiluminescence signal compared to the control, and the LUC/REN ratio was significantly higher (Figure 4B,C). In contrast, the combination of pGreen II-62-SK-*TaERD15L-3B* with pGreen II-0800-LUC-ACGT showed chemiluminescence signal intensity similar to that of the control, with no significant difference in the LUC/REN ratio (Figure 4E,F). These results demonstrated that TaERD15L-3B was capable of binding to the ABRERATCAL *cis*-acting element within the promoter region of the *TaNRX1-2D* gene, thereby positively regulating its expression.

#### 2.2.3. Detection of Transcriptional Activation and Subcellular Localization of TaERD15L-3B as Well as Expression Profile Analysis of TaERD15L-3B Following ABA Treatment

To investigate the transcriptional activation activity of TaERD15L-3B, its full-length CDS was cloned into the pGBKT7 vector and subsequently introduced into Y2HGold yeast competent cells. Both the pGBKT7-*TaERD15L-3B* construct and the empty pGBKT7 vector exhibited normal growth on SD/-Trp solid medium, suggesting that TaERD15L-3B did not exert adverse effects on yeast growth (Figure S3A). However, only the yeast strain carrying pGBKT7-*TaERD15L-3B* was able to grow on SD/-Trp/-His/-Ade solid medium, whereas the yeast strain containing the empty pGBKT7 vector failed to do so (Figure S3A), thereby indicating that TaERD15L-3B possessed transcriptional activation activity.

To determine the subcellular localization of TaERD15L-3B, the protein was fused with GFP and transiently co-expressed with a nuclear marker protein labeled with mCherry in tobacco leaves. Confocal laser microscopy analysis revealed that the GFP signal associated with the TaERD15L-3B fusion protein was localized to the nucleus and cytoplasm, with a comparatively stronger signal observed in the nucleus (Figure S3B).

The expression level of *TaERD15L-3B* began to significantly increase 2 h after treatment with 100 μM ABA and continued to rise after 12 h, suggesting that *TaERD15L-3B* was inducible and upregulated by ABA treatment (Figure S3C).

#### 2.2.4. Silencing of TaERD15L-3B Mediated by BSMV-VIGS Reduced Salt Tolerance in Wheat

**Figure 5:**
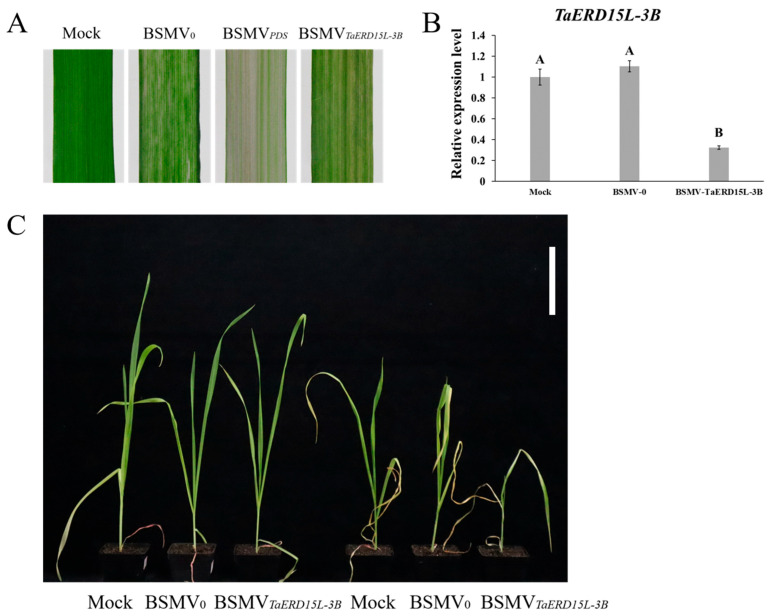
Functional traits of *TaERD15L-3B*-silenced wheat plants under normal and salt stress (200 mM NaCl) conditions: (**A**) Wheat leaves were treated with virus constructs for 10 d. Mock, control inoculations; BSMV_0_, negative control; BSMV*_PDS_*, positive control; BSMV*_TaERD15L-3B_*, BSMV-VIGS mediated *TaERD15L-3B*-silenced wheat plants. (**B**) Gene-silencing efficiency detection after virus-induced silencing. Vertical bars indicate standard deviations. Different capital letters indicate extremely significant differences at *p* < 0.01 within condition according to one-way ANOVA and post hoc Tukey’s test. (**C**) The phenotype analysis of the *TaERD15L-3B*-silenced wheat plants under normal treatment (**left**) and salt stress treatment (**right**, 200 mM NaCl) in wheat. Scale bars: 10 cm.

When tobacco leaves inoculated with BSMV_0_, BSMV*_PDS_*, and BSMV*_TaERD15L-3B_* exhibited obvious disease phenotypes, and the wheat had developed to two-leaf and one-heart stage, the infected tobacco leaves were ground up and then inoculated onto the first leaf of wheat through rubbing. After 14 d, the third leaf of wheat treated solely with PBS buffer remained green. In contrast, the third leaf of the positive control wheat inoculated with BSMV*_PDS_* exhibited symptoms of whitening and chlorosis. Both the negative control wheat inoculated with BSMV_0_ and the experimental group wheat inoculated with BSMV*_TaERD15L-3B_* displayed yellowing stripes on the third leaf, indicating successful virus inoculation (Figure 5A). The expression levels of *TaERD15L-3B* and *TaNRX1-2D* genes were markedly reduced in the BSMV*_TaERD15L-3B_* line compared to those in the Mock and BSMV_0_ lines (Figure 5B and Figure S4). These findings suggested that the BSMV-mediated VIGS system efficiently silenced the expression of the *TaERD15L-3B* gene, which further led to a decrease in the expression level of *TaNRX1-2D*.

**Figure 6:**
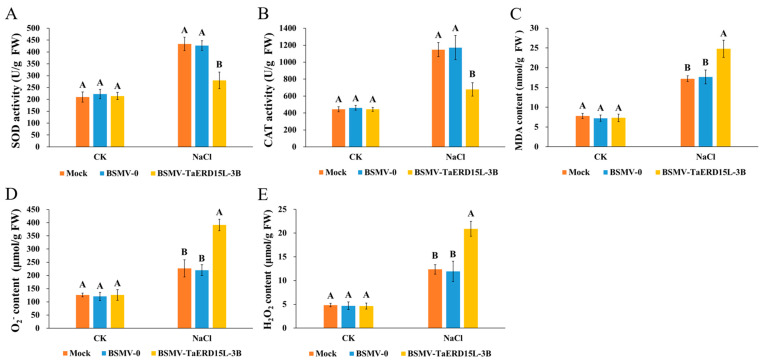
Physiological indices of *TaERD15L-3B*-silenced wheat plants under normal (CK) and salt stress (200 mM NaCl) conditions: (**A**) Enzyme activity of SOD. (**B**) Enzyme activity of CAT. (**C**) MDA content. (**D**) O_2_^−^ content. (**E**) H_2_O_2_ content. Mock, control inoculations; BSMV-0, negative control; BSMV-TaERD15L-3B, BSMV-VIGS mediated *TaERD15L-3B*-silenced wheat plants. Vertical bars indicate standard deviations. Different capital letters indicate extremely significant differences at *p* < 0.01 according to one-way ANOVA and post hoc Tukey’s test.

Following salt stress treatment, wheat leaves inoculated with BSMV*_TaERD15L-3B_* exhibited more severe yellowing and wilting symptoms, whereas those inoculated with Mock and BSMV_0_ showed relatively milder symptoms (Figure 5C). The enzyme activities of SOD and CAT in wheat plants inoculated with BSMV*_TaERD15L-3B_* virus were significantly reduced compared to those in the Mock and BSMV_0_ control lines. Conversely, the levels of O_2_^−^, H_2_O_2_, and MDA were markedly elevated in the BSMV*_TaERD15L-3B_*-inoculated plants (Figure 6). These findings suggested that the *TaERD15L-3B* gene silencing mediated by BSMV-VIGS substantially impaired the antioxidant capacity of wheat, leading to increased oxidative stress and consequently reduced salt tolerance.

### 2.3. Analysis of the Interaction Between TaNRX1-2D and TaCAT2-B

Members of the TRX family, including NRX, typically undergo an instantaneous reduction process when interacting with their target proteins, making it challenging to capture stable complexes. Substitution of one of the cysteine residues within the active center motif “CXXC” with another amino acid can lead to the formation of a stable TRX–target protein complex. TaNRX1-2D possesses two active sites, “CPPC” and “CGPC” [20]. Replacement of the second cysteine residue (“C”) in each of these motifs with serine (“S”) generated the variant protein “TaNRX1-2D (C54, 327S)”.

Kneeshaw et al. [14] identified that the catalases AtCAT1, AtCAT2, and AtCAT3 were target proteins of AtNRX1 in *Arabidopsis* through a “mutation capture strategy”. CAT can efficiently scavenge excess H_2_O_2_ generated under stress conditions, thereby mitigating oxidative damage in plants. According to Zhang et al. [21], salt stress in wheat significantly upregulated the expression of the *TaCAT2-B* (TraesCS6B02G056800) gene. Based on these findings, TaCAT2-B was selected to investigate whether it serves as a target protein of TaNRX1-2D.

**Figure 7:**
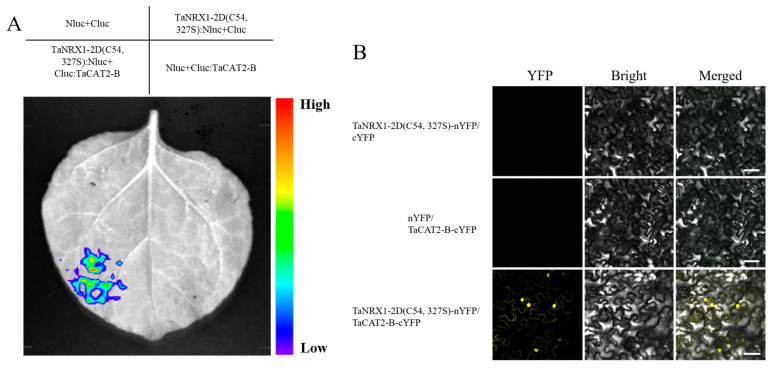
Luciferase complementation assays (LCA) (**A**) and bimolecular fluorescence complementation (BiFC) (**B**) in *N. benthamiana* leaves showed that TaNRX1-2D (C54, 327S) interacted with TaCAT2-B. Scale bars: 30 μm.

The CDS sequence of *TaNRX1-2D* (C54, 327S) lacking a stop codon was cloned into the pCAMBIA1300-NLuc vector to generate the *TaNRX1-2D (C54, 327S)*-NLuc construct. Similarly, the CDS sequence of *TaCAT2-B* was cloned into the pCAMBIA1300-CLuc vector to produce the CLuc-*TaCAT2-B* construct. Four combinations, NLuc+CLuc, *TaNRX1-2D (C54, 327S)*-NLuc+CLuc, NLuc+CLuc-*TaCAT2-B*, and *TaNRX1-2D (C54, 327S)*-NLuc+CLuc-*TaCAT2-B*, were infiltrated into distinct regions of tobacco leaves. Following an adequate dark incubation period, D-luciferin solution was uniformly applied to the infiltration sites as a reaction substrate. The leaves were then placed in a Plant View 100 imaging system to detect chemiluminescence signals. The results demonstrated that the site infiltrated with the *TaNRX1-2D (C54, 327S)*-NLuc+CLuc-*TaCAT2-B* combination exhibited strong chemiluminescence signals, whereas no detectable signals were observed at the sites of the other three control combinations (Figure 7A). These findings indicated a specific interaction between TaNRX1-2D (C54, 327S) and TaCAT2-B.

The CDS sequence of *TaCAT2-B*, excluding the stop codon, was cloned into the pSCY-CE vector to generate the *TaCAT2-B*-cYFP vector. Similarly, the CDS sequence of *TaNRX1-2D (C54, 327S)* was inserted into the pSCY-NE vector to construct the *TaNRX1-2D (C54, 327S)*-nYFP vector. *TaNRX1-2D (C54, 327S)*-nYFP+cYFP and nYFP+*TaCAT2-B*-cYFP were utilized as control groups, whereas *TaNRX1-2D (C54, 327S)*-nYFP and *TaCAT2-B*-cYFP constituted the experimental group. Confocal laser microscopy analysis revealed that tobacco leaves infiltrated with the *TaNRX1-2D (C54, 327S)*-nYFP+*TaCAT2-B*-cYFP combination exhibited clear yellow fluorescence signals. In contrast, no yellow fluorescence was detected in tobacco leaves infiltrated with the control combination (Figure 7B). These findings suggested that TaNRX1-2D (C54, 327S) was capable of interacting with TaCAT2-B.

### 2.4. Proteomic Analysis of TaNRX1-2D Transgenic Wheat Under Salt Stress

#### 2.4.1. Quality Assessment of Proteomics

All 18 wheat leaf samples successfully passed the total protein quality assessment and were suitable for subsequent protein sequencing analysis (Table S3).

A total of 72,953 peptide segments were identified, with 11,122 proteins matched in wheat leaf samples (Figure S5). The comparable number of proteins identified across different biological replicates within the same treatment suggested that the protein identification and quantification methods employed in this study were highly effective, enabling comprehensive proteomic coverage and precise quantification in wheat leaves.

Based on the violin plot depicting the distribution of CV values from protein quantification across samples, it was evident that the CV values among biological replicates were generally low (Figure S6), indicating a high level of sample repeatability.

#### 2.4.2. Differentially Expressed Protein (DEP) Analysis

**Figure 8:**
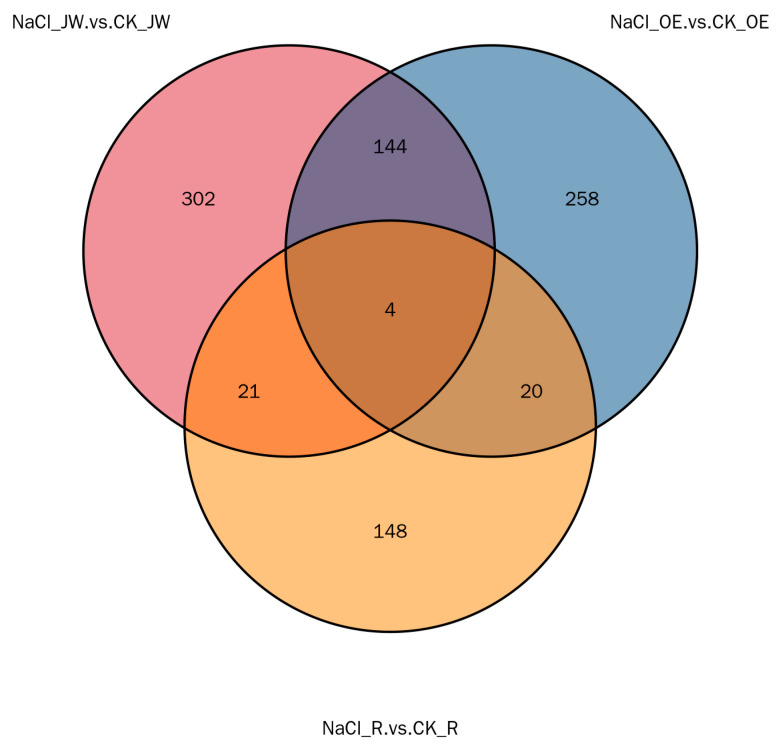
Venn diagram of differentially expressed proteins in JW (WT) and *TaNRX1-2D* transgenic wheat lines before and after salt stress. CK_JW, JW wheat variety (WT) under the normal condition. CK_OE, *TaNRX1-2D* overexpression in transgenic wheat under normal condition. CK_R, *TaNRX1-2D* RNA interference transgenic wheat under normal condition. NaCl_JW, JW wheat variety (WT) under the salt stress (200 mM NaCl) condition. NaCl_OE, *TaNRX1-2D* overexpression transgenic wheat under the salt stress (200 mM NaCl) condition. NaCl_R, *TaNRX1-2D* RNA interference transgenic wheat under the salt stress (200 mM NaCl) condition.

To identify DEPs specifically influenced by *TaNRX1-2D*, we analyzed the intersection of DEPs from both the *TaNRX1-2D*-OE line and the RNAi line before and after salt stress treatment (Figure 8). Subsequently, DEPs commonly shared with JW (WT) within this intersection were excluded. Ultimately, 20 DEPs specifically affected by *TaNRX1-2D* were identified. These proteins included endochitinase, hexokinase, non-specific lipid-transfer protein, thaumatin, aldo-keto reductase, polyol transporter, amidophosphoribosyltransferase, glucan endo-1,3-beta-glucosidase, THO complex subunit, transmembrane protein, cinnamoyl-CoA reductase, lectin-domain containing receptor kinase, wheatwin-1 precursor, pathogenesis-related protein, ribulose-1,5-bisphosphate carboxylase/oxygenase, and three uncharacterized proteins (Table S4).

## 3. Discussion

### 3.1. TaNRX1-2D Positively Regulates Salt Stress Tolerance in Transgenic Wheat (Triticum aestivum *L.*)

In recent years, climate and environmental changes have led to a gradual increase in soil salt content, making salt stress one of the major environmental factors that constrain global agricultural production and pose a serious threat to staple crops such as wheat [3,22]. Salt stress can induce excessive accumulation of ROS in plants, thereby causing severe oxidative damage [18,23]. Plants have developed diverse antioxidant defense systems to counteract the detrimental effects of adverse stress. The redox reactions facilitated by members of the TRX family represent crucial molecular mechanisms that safeguard target proteins, including a variety of enzymes, against oxidative damage. NRX may play a pivotal role in this protective mechanism [14,15,24]. Although the NRX has been demonstrated to enhance stress resistance in certain plant species, and our previous studies have indicated that the *TaNRX1-2D* gene in wheat might positively regulates drought tolerance through modulating antioxidant enzyme activity, proline and soluble sugar content, as well as photosynthetic efficiency [17], its functional role and underlying molecular regulatory mechanisms in wheat’s response to salt stress remain to be further elucidated. In this study, under salt stress conditions, overexpression of *TaNRX1-2D* led to higher survival rates of wheat in both the seedling and heading stages compared to WT, whereas RNAi lines exhibited reduced survival rates, suggesting that the *TaNRX1-2D* gene positively regulated salt tolerance in wheat. Under salt stress, excessive accumulation of ROS, such as O_2_^−^ and H_2_O_2_, can cause oxidative damage to cellular components, including nucleic acids, proteins, and lipids, thereby impairing normal physiological functions. Within the ROS scavenging system, SOD catalyzes the dismutation of O_2_^−^ into O_2_ and H_2_O_2_, which is subsequently decomposed into H_2_O and O_2_ by CAT [21,25]. Following salt stress treatment, the *TaNRX1-2D*-OE lines exhibited significantly higher SOD and CAT enzyme activities than the WT, resulting in lower levels of O_2_^−^ and H_2_O_2_. In contrast, RNAi lines displayed the opposite trend, further supporting the role of *TaNRX1-2D* in enhancing wheat salt tolerance through its involvement in ROS-scavenging pathways.

### 3.2. TaERD15L-3B Is an Upstream Regulatory Factor of TaNRX1-2D

The ABA signaling pathway plays a crucial role in plant responses to environmental stress and adversity. Salt stress, which results in an imbalance of ion concentrations inside and outside plant cells, can trigger intracellular osmotic stress signals, leading to the accumulation of ABA. ROS are involved in the stress-induced accumulation of ABA. As a key signaling molecule, ABA further mediates the expression of genes associated with salt stress responses [18]. Previous research in our laboratory identified that a 36-bp fragment (−193 bp to −157 bp) within the *TaNRX1-2D* gene promoter served as a critical regulatory region in response to ABA signaling. This fragment contains two *cis*-acting elements associated with ABA-responsive pathways: ABRERATCAL and ACGTATERD1 [19]. This study demonstrated that TaERD15L-3B was capable of binding to the ABRERATCAL *cis*-acting element and positively regulating the expression of the *TaNRX1-2D* gene. The *ERD* gene family was originally named based on its members’ ability to be rapidly induced under dehydration stress [26]. ERD15, a hydrophilic protein within the ERD family, contained a PAM2 domain and was exclusively found in plants [27]. GmERD15 in soybean has been identified as a novel transcription factor that responds to both ER stress and osmotic stress. This protein was localized in both the nucleus and cytoplasm. It was able to bind to the promoter region of the *NRP-B* gene and positively regulate its expression. The cell death signaling pathway mediated by the GmERD15-*NRP-B* module was hypothesized to play a role in soybean’s adaptive response to various stress conditions [28]. After the overexpression of *SpERD15* in *Solanum pennellii* in tobacco, the overexpression lines exhibited enhanced tolerance to drought and salt stress, whereas the *SpERD15* suppression lines displayed reduced tolerance to these stress conditions [29]. The promoters of *ERD15* family members in maize were found to contain numerous *cis*-acting elements associated with stress and hormone responses. *ZmERD15c* and *ZmERD15d* were upregulated under salt stress conditions, and their heterologous overexpression in yeast significantly improved salt stress tolerance [30]. Similar to previous research findings, the *TaERD15L-3B* gene was found to be upregulated under ABA and salt stress inductions. The TaERD15L-3B protein was localized in the nucleus and exhibited transcriptional activation activity, suggesting that TaERD15L-3B functioned as a transcription factor that was enriched under salt stress and played a role in the ABA signaling pathway. When *TaERD15L-3B* was silenced in wheat using the BSMV-mediated VIGS system, the silenced lines displayed a significantly reduced capacity to scavenge ROS under salt stress, a phenotype comparable to that observed in *TaNRX1-2D* RNAi lines under similar conditions. These results indicated that TaERD15L-3B acted as a positive regulator of salt tolerance in wheat by modulating the expression of *TaNRX1-2D*.

### 3.3. TaCAT2-B Is the Target Protein of TaNRX1-2D

Members of the TRX superfamily typically contain at least one Cys-X-X-Cys (CXXC) catalytic motif, in which one cysteine serves as the catalytic residue, while the other usually functions as a resolving residue [15]. The catalytic cysteine residue typically forms a transient mixed disulfide bond with one of the cysteine residues in its target protein. If the resolving cysteine residue is mutated to serine or alanine, the thiol–disulfide exchange process is interrupted, resulting in the stabilization of the mixed disulfide intermediate. This stabilization leads to the formation of a persistent interaction between the TRX protein and its target protein, thereby generating what is referred to as an “interacting protein” [16]. NRX typically contains two CXXC catalytic motifs, through which it cooperatively or sequentially reduces target proteins, a process referred to as the “dicysteinic mechanism” [14]. Therefore, in this study, we introduced a mutation substituting the second cysteine residue in both active sites of TaNRX1-2D with serine to investigate its potential interaction with the wheat CAT protein. CAT is a widely conserved antioxidase found across organisms, capable of efficiently catalyzing the decomposition of H_2_O_2_ into H_2_O and O_2_. As a key component of the cellular defense system against oxidative stress, CAT plays a crucial role in maintaining metabolic homeostasis within organisms [21]. All members of the CAT family in *Arabidopsis*, including CAT1, CAT2, and CAT3, were identified as target proteins of AtNRX1 [14]. During the process of H_2_O_2_ scavenging, *Arabidopsis* CAT experienced oxidative damage. NRX interacted with and reduced CAT via a thiol-disulfide exchange mechanism, thereby restoring its enzymatic activity [14]. In this study, following salt stress treatment in wheat, the CAT enzyme activity in *TaNRX1-2D*-OE lines was significantly higher than that in the WT, whereas the H_2_O_2_ content was lower. Conversely, the *TaNRX1-2D*-RNAi lines exhibited reduced CAT activity and elevated H_2_O_2_ levels. These findings suggested that TaNRX1-2D may regulate CAT activity in wheat, potentially through direct targeting. When wheat was exposed to salt stress, the expression abundance of *TaCAT2-B* was significantly upregulated [21]. Therefore, TaCAT2-B was selected to verify its potential interaction with TaNRX1-2D as a putative target protein. The results of the BiFC assay in this study demonstrated that TaNRX1-2D (C54, 327S) interacted with TaCAT2-B both in the nucleus and cytoplasm. Based on these findings, we hypothesized that TaNRX1-2D may target TaCAT2-B in wheat, reduce incorrectly formed disulfide bonds, and thereby restore its correct spatial conformation and catalytic activity, ultimately enhancing the capacity to detoxify H_2_O_2_ in wheat.

### 3.4. TaNRX1-2D Mediates Salt Stress Tolerance in Wheat by Regulating the Protein Abundance of Hexokinase (HXK) and Non-Specific Lipid-Transfer Protein (nsLTP)

Based on the aforementioned results, certain aspects of the molecular mechanism through which *TaNRX1-2D* enhances salt tolerance in wheat have been elucidated. However, the comprehensive molecular pathways by which *TaNRX1-2D* contributes to salt tolerance in wheat remain largely unclear. In this study, potential molecular mechanisms underlying *TaNRX1-2D*-mediated salt tolerance in wheat were investigated using a DIA-based proteomic approach. This study identified a total of 20 DEPs that were specifically regulated by *TaNRX1-2D* before and after salt stress treatment in wheat. Among these 20 DEPs, hexokinase and nsLTP have been extensively investigated for their regulatory roles in plant responses to abiotic stress. Following salt stress, the protein abundance of hexokinase TaHXK7-1A (TraesCS1A02G122800) was observed to decrease in the OE line, whereas it increased in the RNAi line (Table S4). The three members of the wheat nsLTP family located on chromosomes 1B (TraesCS1B02G394500), 5D (TraesCS5D02G145300), and 7B (TraesCS7B02G351900) exhibited increased protein abundance in the OE line and reduced abundance in the RNAi line (Table S4). These findings suggested that hexokinase TaHXK7-1A and the three nsLTP proteins in wheat may be regulated by TaNRX1-2D to mediate salt tolerance in wheat.

Hexokinase typically exhibits dual functionalities, catalyzing the phosphorylation of hexoses and acting as a glucose sensor that responds to environmental changes by detecting glucose signals [31,32]. Glucose functions not only as an energy source but also as a signaling molecule. Global transcriptome analyses and numerous pharmacological studies have demonstrated that a variety of plant genes are regulated by glucose signaling pathways [33]. Osmotic stresses, such as those caused by salinity and drought, can lead to an increase in glucose levels, and glucose signaling has been shown to promote the biosynthesis of ABA [34]. However, excessive accumulation of glucose may negatively affect photosynthesis and impede plant growth [35]. Tang et al. [34] observed that transgenic *Arabidopsis* with heterologous overexpression of *TaHXK7-1A* exhibited significantly elevated levels of ROS when grown on MS medium supplemented with 6% glucose, compared to the WT. This increase in ROS ultimately resulted in leaf chlorosis and delayed growth. Respiratory burst oxidase homolog (RBOH) is recognized as a key enzyme responsible for ROS production in plants. Under drought stress conditions, *RBOH* gene expression in *TaHXK7-1A*-OE transgenic *Arabidopsis* was markedly upregulated, leading to excessive accumulation of ROS and MDA, which consequently impaired drought tolerance and reduced survival rates. In contrast, wheat lines with *TaHXK7-1A* gene silencing mediated by BSMV-VIGS showed decreased expression of *RBOH* genes, reduced ROS accumulation, and enhanced drought resistance. Based on these findings, it is hypothesized that *TaHXK7-1A* may enhance the expression of wheat *RBOH* genes through glucose signaling pathways, thereby inducing excessive ROS production and causing significant oxidative damage to wheat plants. The regulatory pathway involving *TaNRX1-2D* can decrease the protein abundance of TaHXK7-1A, thereby mitigating oxidative damage in wheat under salt stress conditions.

The plant nsLTP protein facilitates the transfer of lipid substances between cell membranes, thereby playing a crucial role in plant growth, development, and stress response mechanisms [36]. Transgenic tobacco overexpressing the *NtLTPI.38* gene exhibited reduced chlorophyll degradation, enhanced osmotic regulation, and increased antioxidant capacity, leading to improved heat tolerance compared to the control group under heat stress conditions [37]. In cotton, the *GhLTP4* gene was significantly upregulated following treatment with ABA and exposure to drought stress. Transgenic cotton lines overexpressing *GhLTP4* demonstrated elevated ABA levels and alterations in both the composition and concentration of lipid metabolites in response to drought stress, as compared to the WT. Furthermore, the expression levels of genes encoding key enzymes involved in the tricarboxylic acid (TCA) cycle were increased, resulting in higher ATP content in transgenic cotton leaves and consequently enhanced drought resistance [38]. The *GmLTPI.3* gene in soybean was significantly upregulated under drought and salt stress conditions. Overexpression of the *GmLTPI.3* gene in soybean enhanced the expression of *GmSOD1* and *GmSOD2*, while reducing the expression levels of *GmRBOHA* and *GmRBOHB*, two ROS-producing-related genes. This led to a significant decrease in root ROS accumulation and ultimately improved the tolerance of transgenic plants to drought and salt stress [39]. Based on these findings, we hypothesized that the regulatory pathway involving *TaNRX1-2D* may enhance the protein abundance of nsLTP in wheat. Under salt stress, this mechanism could improve lipid and energy metabolism in wheat, thereby enhancing osmotic regulation and antioxidant capacity, ultimately contributing to improved salt tolerance. Further research is required to elucidate the specific molecular mechanisms involved.

## 4. Materials and Methods

### 4.1. Plant Materials and Stress Treatments

To analyze the function of *TaNRX1-2D* in salt stress tolerance of wheat at seedling stage, the seeds of WT (“JW”, a spring common wheat variety which selected from a segregating population of the cross of spring common wheat varieties “Fielder” and “NB1”) and *TaNRX1-2D* transgenic wheat (*TaNRX1-2D*-OE-1, *TaNRX1-2D*-OE-3, *TaNRX1-2D*-OE-6, *TaNRX1-2D*-RNAi-6, *TaNRX1-2D*-RNAi-8, and *TaNRX1-2D*-RNAi-9) preserved in our laboratory [17] were sown in pots containing the same volume of nutrient soil (4 plants per pot) and cultivated in light incubator (16-h-light, 25 °C/8-h-dark, 20 °C, 70% humidity, and 18,000 Lux light intensity). When the wheat plants reached the three-leaf and one-heart stage, the control plants were watered normally, and the other wheat plants were treated with a 200 mM NaCl solution. The physiological indexes were measured 7 d after salt stress treatment, and the phenotype and survival rates were recorded 14 d later.

To analyze the function of *TaNRX1-2D* in salt stress tolerance of wheat at heading stage, WT and *TaNRX1-2D* transgenic wheat seeds were sown in pots containing the same volume of nutrient soil (8 plants per pot) and cultivated in a greenhouse (16 h light, 25 °C/8 h dark, 18 °C). When the apex of the primary spike emerged from the sheath of the flag leaf, the control plants were watered normally, and the other wheat plants were treated with a 200 mM NaCl solution. The physiological indexes were measured 10 d after salt stress treatment, and the phenotype and survival rates were recorded 20 d later.

### 4.2. Physiological and Biochemical Index Analyses

The superoxide dismutase (SOD) activity, catalase (CAT) activity, malondialdehyde (MDA) content, hydrogen peroxide (H_2_O_2_) level and superoxideanion (O_2_^−^) level were detected using the Solarbio assay kits (BC5165 for SOD; BC0205 for CAT; BC0025 for MDA; BC3595 for H_2_O_2_; and BC1295 for O_2_^−^) according to the manufacturer’s instructions. These experiments were conducted in triplicate, with seven biological replicates assigned to each group.

### 4.3. Yeast One-Hybrid Assay

Previous research conducted in our laboratory has identified a 36 bp fragment (−193 bp to −157 bp, with the translation initiation site “ATG” designated as position 0) within the *TaNRX1-2D* gene promoter as a critical regulatory region responsive to ABA signaling [19]. This fragment contains two *cis*-acting elements associated with ABA signaling: ABRERATCAL (“CACGCGG”, abbreviated as ABRE) and ACGTATERD1 (“ACGT”, abbreviated as ACGT). The sequence “CCTCACGCGGTCACGTCCC” containing the above two *cis*-elements and flanking sequences was repeated thrice and then inserted into the pAbAi vector with the homologous recombination method to construct the pAbAi-(ABRE+ACGT) recombinant vector. The restriction enzymes used were *Sac*I and *Sal*I. All primers used in the experiments are listed in Table S1. The pAbAi-(ABRE+ACGT) recombinant vectors were introduced into Y1HGold yeast competent cells (Protein Interaction Bio., Wuhan, China), and the transformed cells were subsequently cultured on SD/-Ura solid medium at 30 °C supplemented with 0, 200, 400, 600, 800, and 1000 ng/mL Aureobasidin A (AbA, Coolaber, Beijing, China) to determine the minimum inhibitory concentration.

The pAbAi-(ABRE+ACGT) yeast cells were prepared into competent cells using the EX-Yeast Transformation Kit (Zoman Biotechnology, Beijing, China). Subsequently, the wheat cDNA library plasmids preserved in the laboratory were introduced into pAbAi-(ABRE+ACGT) yeast competent cells through transformation, followed by cultivation on SD/-Ura/-Leu solid medium supplemented with AbA at the minimum inhibitory concentration at 30 °C for screening purposes. Colony growth was observed after 3–5 d, and all monoclones identified through colony PCR were subsequently sequenced for further identification (Tsingke, Beijing, China).

The coding sequences (CDS) of the candidate transcription factor genes obtained from the aforementioned study were inserted into the pGADT7 vector via homologous recombination to generate the pGADT7-prey construct, using the restriction enzymes *EcoR*I and *Bam*HI. pAbAi-(ABRE+ACGT) and pGADT7-prey were co-transformed into Y1HGold yeast competent cells, which were then cultured on SD/-Ura/-Leu solid medium, either with or without the minimum inhibitory concentration of AbA at 30 °C, to validate the yeast one-hybrid screening results in a point-to-point manner. Colony formation was monitored and documented after an incubation period of 3–5 d.

### 4.4. Dual-Luciferase Reporter Assay

The full-length CDS of *TaERD15L-3B* was inserted into the pGreen II 62-SK vector as the effector vector using *Bam*HI and *Xho*I restriction enzymes. The sequences consisting of three consecutive repetitions of “ABRE+ACGT”, “ABRE”, and “ACGT” were separately inserted into the pGreen II 0800-LUC vector as the three reporter vectors using *Kpn*I and *Xho*I restriction enzymes. Agrobacterium tumefaciens strain GV3101 (pSoup-19) (AngYu Biotechnologies, Shanghai, China) carrying effector vector and reporter vector were co-infiltrated into *N. benthamiana* leaves. For the qualitative assay, following 48 h of incubation at 25 °C, 1 mM D-luciferin solution (Coolaber, Beijing, China) was applied to designated positions on the leaves of *N. benthamiana*. At least six independent leaves were used for each experimental replicate. The leaves were then incubated in darkness for 5–10 min. LUC signals were detected using a low-light cooled charge-coupled device (CCD) imaging apparatus, PlantView100 (Biolight, Guangzhou, China). For the quantitative assay, transfected *N. benthamiana* leaf samples were collected using a puncher and subsequently lysed using a Firefly and Renilla Dual Luciferase Assay Kit (UE Landy, Suzhou, China). The activities of LUC (firefly luciferase) and REN (Renilla luciferase) were measured separately using a Multi-Mode Microplate Reader (Tecan Group, Sydney, Austria). The LUC/REN ratio was calculated to evaluate the relative luciferase activity.

### 4.5. RNA Extraction and Real-Time Quantitative PCR (RT-qPCR)

Total RNA was extracted using TRNzol reagent (TIANGEN, Beijing, China), and the isolated RNA was subsequently used as a template for cDNA synthesis through a reverse transcription kit (TransGen, Beijing, China). The ABI Life Fluorescence Quantitative PCR System Q3 was used to perform RT-qPCR. The 20 µL reactive system contained 10-µL ArtiCanCEO SYBR qPCR Mix (Tsingke, Beijing, China), 0.4 µL forward and reverse primers for each (0.2 μM), 1 µL cDNA (200 ng/μL), 7.8 µL nuclease-free water, and 0.4 µL ROX Reference Dye II (50×). The reaction conditions were 95 °C for 5 min, 40 cycles of 95 °C for 10 s, and 60 °C for 30 s, according to the manufacturer’s protocols. All of the specific primers are listed in Table S1. The 2^−ΔΔCt^ method was used to calculate the gene transcriptional abundance [40], and the wheat *β-actin* gene was used as the reference gene.

### 4.6. Transcriptional Activation Analysis of TaERD15L-3B Protein

For the transactivation assay, the CDS fragment of *TaERD15L-3B* was inserted into the pGBKT7 vector using *Eco*RI and *Sal*I restriction enzymes. The recombinant plasmid and the control empty vector (pGBKT7) were individually introduced into Y2HGold yeast competent cells (Protein Interaction Bio., Wuhan, China) and cultured on SD/-Trp solid medium at 30 °C. Subsequently, 1 μL of monoclonal yeast solution (OD600 = 1.0) was inoculated onto SD/-Trp and SD/-Trp/-His/-Ade solid media and incubated at 30 °C. Colony formation was monitored and documented after an incubation period of 3–5 d.

### 4.7. Subcellular Localization Assay of TaERD15L-3B

The CDS without the termination codon of *TaERD15L-3B* was inserted into the pCAMBIA1302-GFP vector using the *Bst*BI restriction enzyme. *Agrobacterium tumefaciens* strain GV3101 (Tsingke, Beijing, China) carrying the fusion vector was infiltrated into *N. benthamiana* leaves. After incubation for 48 h at 25 °C, the corresponding fluorescence signals in *N. benthamiana* leaves were captured using a laser confocal fluorescence microscope FV3000 (Olympus, Tokyo, Japan).

### 4.8. Gene Silencing of TaERD15L-3B in Wheat

Common wheat is allohexaploid and typically possesses three copies of most genes. Due to the high sequence similarity in the coding regions among these three copies, there is a significant degree of functional complementarity, which complicates the analysis of gene function through single-copy silencing approaches [41]. This functional redundancy can be mitigated by simultaneously silencing the target gene along with its homologous counterparts. In this study, a 140 bp conserved cDNA fragment (+295 bp to +434 bp, with the translation initiation site “ATG” designated as position +1) from *TaERD15L-3B* was cloned and introduced into pCaBS-γ to produce the BSMV-*TaERD15L-3B* construct, which would potentially lead to the simultaneous silencing of *TaERD15L-3B* along with its corresponding homologous gene cluster members (Figure S1). The construction of the recombinant vector and its application in the VIGS system were carried out in accordance with the method of Yuan et al. [42]. Two-leaf stages of the wheat variety Xinong 20 (XN 20) were inoculated with the BSMV-*TaERD15L-3B* construct. Phosphate-buffered saline was used as the mock control, BSMV-PDS as the positive control, and BSMV0 as the negative control.

Following a 10-day inoculation period, the presence of symptoms was confirmed and captured using an SZX16 stereoscopic microscope (Olympus, Tokyo, Japan). The transcript levels of *TaERD15L-3B* and *TaNRX1-2D* were then evaluated using RT-qPCR. Subsequently, *TaERD15L-3B*-silenced wheat plants were subjected to 200 mM NaCl treatment for 20 d to evaluate their phenotypic responses.

Following 20 d of salt stress treatment, stress-related physiological indices were evaluated in Mock, BSMV_0_, and BSMV*_TaERD15L-3B_* plants. The methods for measuring physiological indices were described in Section 2.2.

### 4.9. The Luciferase Complementation Assay (LCA)

LCA was conducted in accordance with the method outlined by Chen et al. [43]. The CDS without the termination codon of *TaNRX1-2D (C54, 327S)* and the full-length CDS of *TaCAT2-B* were individually inserted into the pCAMBIA1300-Nluc and pCAMBIA1300-Cluc vectors using *Kpn*I and *Sal*I restriction enzymes. The *Agrobacterium tumefaciens* strain GV3101 (Tsingke, Beijing, China) carrying different recombinant plasmids was co-infiltrated into *N. benthamiana* leaves. Following 48 h of incubation at 25 °C, 1 mM D-luciferin solution (Coolaber, Beijing, China) was applied to designated positions on the leaves of *N. benthamiana*. At least six independent leaves were used for each experimental replicate. The leaves were then incubated in darkness for 5–10 min. LUC signals were detected using a low-light cooled charge-coupled device (CCD) imaging apparatus, PlantView100 (Biolight, Guangzhou, China).

### 4.10. The Bimolecular Fluorescence Complementation (BiFC) Assay

The full-length CDS of *TaNRX1-2D (C54, 327S)* and the CDS without the termination codon of *TaCAT2-B* were individually inserted into the pSCY-NE and pSCY-CE vectors using *Bam*HI/*Sal*I and *Bam*HI/*Cla*I restriction enzymes. *Agrobacterium tumefaciens* strain GV3101 (Tsingke, Beijing, China) carrying different recombinant plasmids was co-infiltrated into *N. benthamiana* leaves. After incubation for 48 h at 25 °C, the corresponding fluorescence signals in *N. benthamiana* leaves were captured using a laser confocal fluorescence microscope FV3000 (Olympus, Tokyo, Japan).

### 4.11. Data-Independent Acquisition (DIA) Proteomics

The wheat leaf samples utilized for proteomic analysis (Novogene, Beijing, China) were collected from *TaNRX1-2D*-OE, *TaNRX1-2D*-RNAi, and WT wheat plants at the three-leaf and one-heart stage, under both normal conditions and after 7 d of salt stress treatment, as described in Section 2.1. The protein samples were first dissolved with DB lysis buffer (6M Urea, 100 mM TEAB, pH 8.5). Then, the protein solution, trypsin, and 100 mM TEAB buffer were mixed well and digested at 37 °C for 4 h. The pH of the digested samples was adjusted to less than 3 with formic acid, and centrifugation (4 °C, 12,000 *g*, 5 min) was performed. The supernatant was slowly loaded onto the C18 desalting column, and then the eluents of each sample were lyophilized.

The lyophilized powder was dissolved with 10 μL mobile phase (99.9% ddH_2_O, 0.1% formic acid); then, centrifugation (4 °C, 14,000 *g*, 20 min) was performed, and the supernatant samples were subjected to liquid-quality detection. A Thermo Orbitrap Astral mass spectrometer was used with a Vanquish Neo upgraded UHPLC system. The acquisition mode was data-independent. The first-stage mass spectrometry scanning range was set to *m/z* 380–980. The parent ion window size was set to 2-Th, and the number of DIA windows was set to 300. The secondary acquisition range was set to *m/z* 150–2000, and the sub-ion resolution was set to 80,000. The mass spectrometry detection raw data were then output.

DIA-NN library search software was used to analyze the raw data according to the wheat protein database (*Triticum_aestivum*_L_uniprot_2024_07_26.fasta). The mass deviation of precursor ions and fragment ions was automatically detected and corrected. To improve the quality of analysis results, the DIA-NN library search software further filters the search results, and only peptides with a Global.Q.Value < 0.01 and proteins with a PG.Q.Value < 0.01 were retained. Protein quantification results were statistically analyzed using the *t*-test, and proteins with significant quantitative differences (*p* < 0.05, FC > 2.0 or FC < 0.5) between the experimental group and the control group were defined as differentially expressed proteins (DEPs). The BLAST tool was utilized within the NCBI database to obtain annotation information for the DEPs.

The mass spectrometry proteomics data have been deposited to the ProteomeXchange Consortium (https://proteomecentral.proteomexchange.org (accessed on 5 August 2025)) via the iProX partner repository [44,45] with the dataset identifier PXD066958.

### 4.12. Statistical Analysis

All experiments were independently repeated at least thrice. SPSS software (version 22.0; SPSS, Chicago, IL, USA) was used for statistical analysis. One-way analysis of variance and least significant difference post hoc test were used for data analysis.

## 5. Conclusions

In summary, we demonstrated that *TaNRX1-2D* positively regulated salt tolerance in wheat. The TaERD15L-3B transcription factor, whose expression can be induced by NaCl and ABA, positively regulated the expression of *TaNRX1-2D* by specifically binding to the ABRERATCAL *cis*-acting element located in the promoter region of *TaNRX1-2D*. TaNRX1-2D interacted with the H_2_O_2_-scavenging enzyme TaCAT2-B, leading to an enhancement of its enzymatic activity. DIA proteomics analysis suggested that *TaNRX1-2D* may contribute to salt tolerance in wheat by positively regulating the abundance of nsLTP proteins and negatively regulating the abundance of hexokinase. This study not only enhances the understanding of the signal transduction pathways and physiological responses in wheat under salt stress, but also identifies a promising genetic target for engineering salt-tolerant wheat germplasm.

## Data Availability

The data supporting the findings of this study are available from the corresponding author upon request.

## Supplementary Materials

The following supporting information can be downloaded at: https://www.mdpi.com/article/10.3390/plants15010146/s1, Figure S1: Multiple-sequence alignment of *TaERD15L-3A/3B/3D* genes; Figure S2: Relative expression levels of *TaERD15L-3B* and *TabHLH-6D* in response to NaCl (200 mM) for 0 h, 0.5 h, 1 h, 2 h, 6 h, 12 h, 24 h and 48 h in the leaves of wheat variety Chinese Spring at the two-leaf stage; Figure S3: The transcription-activity analysis (A) and subcellular localization (B) of TaERD15L-3B, as well as the relative expression level of *TaERD15L-3B* in response to ABA treatment (100 μM) for 0 h, 0.5 h, 1 h, 2 h, 6 h, 12 h, 24 h and 48 h in the leaves of wheat variety Chinese Spring at the two-leaf stage (C); Figure S4: The relative expression level of *TaNRX1-2D* after virus induced silencing of *TaERD15L-3B*; Figure S5: The number of proteins identified in different samples; Figure S6: The violin plot of the coefficient of variation in protein quantity across different sample replicates; Table S1: Information of primers used in this study; Table S2: Information of candidate transcription factors; Table S3: Protein quality assessment results; Table S4: Identification of differentially expressed proteins in leaves of *TaNRX1-2D* transgenic and WT wheat plants.
