# Supplementary material for: The Wheat Nucleoredoxin TaNRX1-2D Gene Ameliorates Salt Tolerance in Wheat (Triticum aestivum L.)
Source: Plants (Basel). 2026 Jan 4;15(1):146. doi: 10.3390/plants15010146 (PMC12787453; doi:10.3390/plants15010146)
Supplement: Supplementary file 1 [file plants-15-00146-s001.zip › Supplemental Figures.pdf]

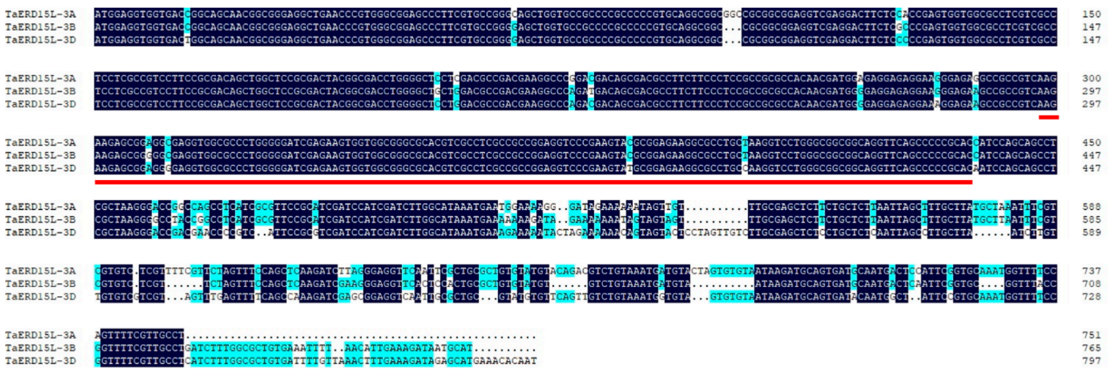

Figure S1. Multiple-sequence alignment of *TaERD15L-3A/3B/3D* genes. The red lines indicated the conserved cDNA segments used for VIGS.

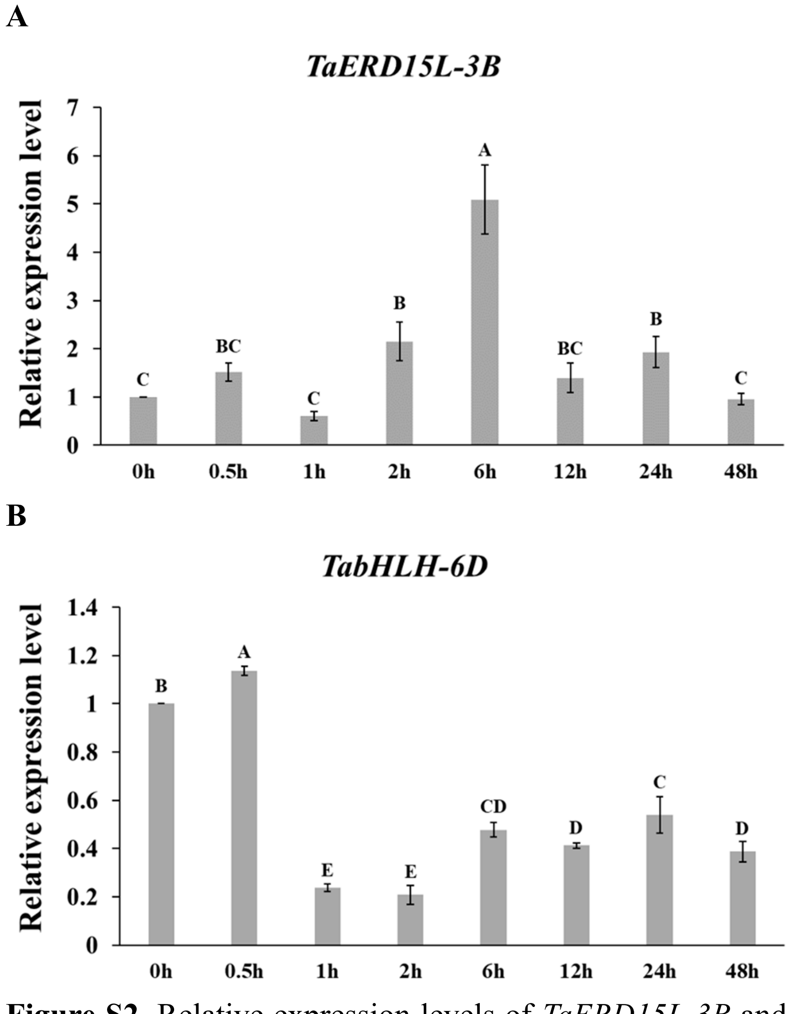

Figure S2. Relative expression levels of *TaERD15L-3B* and *TabHLH-6D* in response to NaCl (200 mM) for 0 h, 0.5 h, 1 h, 2 h, 6 h, 12 h, 24 h and 48 h in the leaves of wheat variety Chinese Spring at the two-leaf stage. Data were normalized with *β-actin* gene of wheat. Vertical bars indicate standard deviations. Different capital letters indicated extremely significant differences at $p < 0.01$ according to one-way ANOVA and post-hoc Tukey's test.

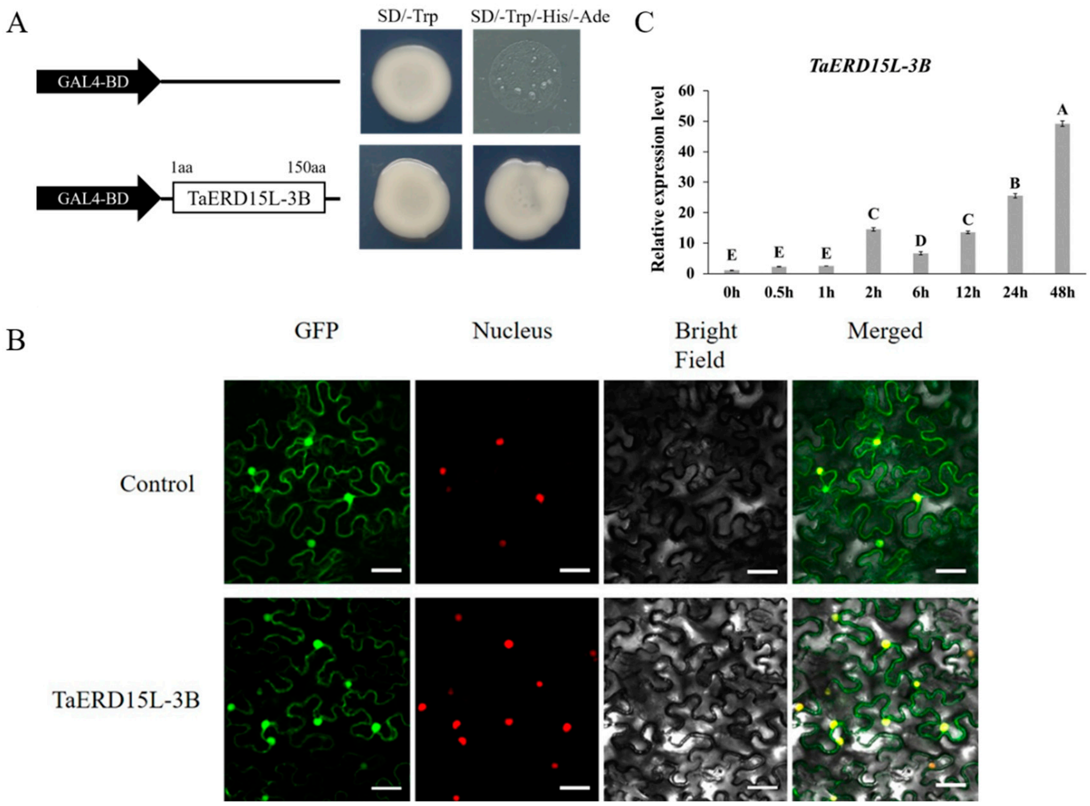

Figure S3. The transcription-activity analysis (A) and subcellular localization (B) of TaERD15L-3B, as well as the relative expression level of *TaERD15L-3B* in response to ABA treatment (100 μM) for 0 h, 0.5 h, 1 h, 2 h, 6 h, 12 h, 24 h and 48 h in the leaves of wheat variety Chinese Spring at the two-leaf stage (C). Scale bars: 30 μm. The gene expression data were normalized with *β-actin* gene of wheat. Vertical bars indicated standard deviations. Different capital letters indicated extremely significant differences at $p < 0.01$ according to one-way ANOVA and post-hoc Tukey's test.

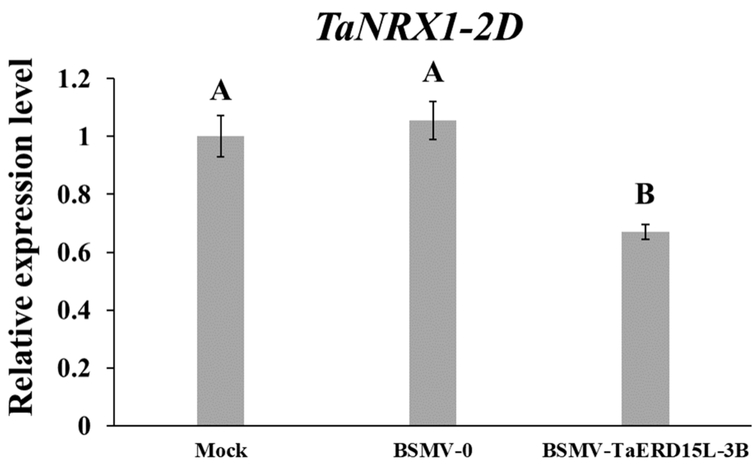

Figure S4. The relative expression level of *TaNRX1-2D* after virus induced silencing of *TaERD15L-3B*. Mock, control inoculations; BSMV-0, negative control; BSMV-TaERD15L-3B, BSMV-VIGS mediated *TaERD15L-3B*-silenced wheat plants. Vertical bars indicated standard deviations. Different capital letters indicated extremely

significant differences at $p < 0.01$ according to one-way ANOVA and post-hoc Tukey's test.

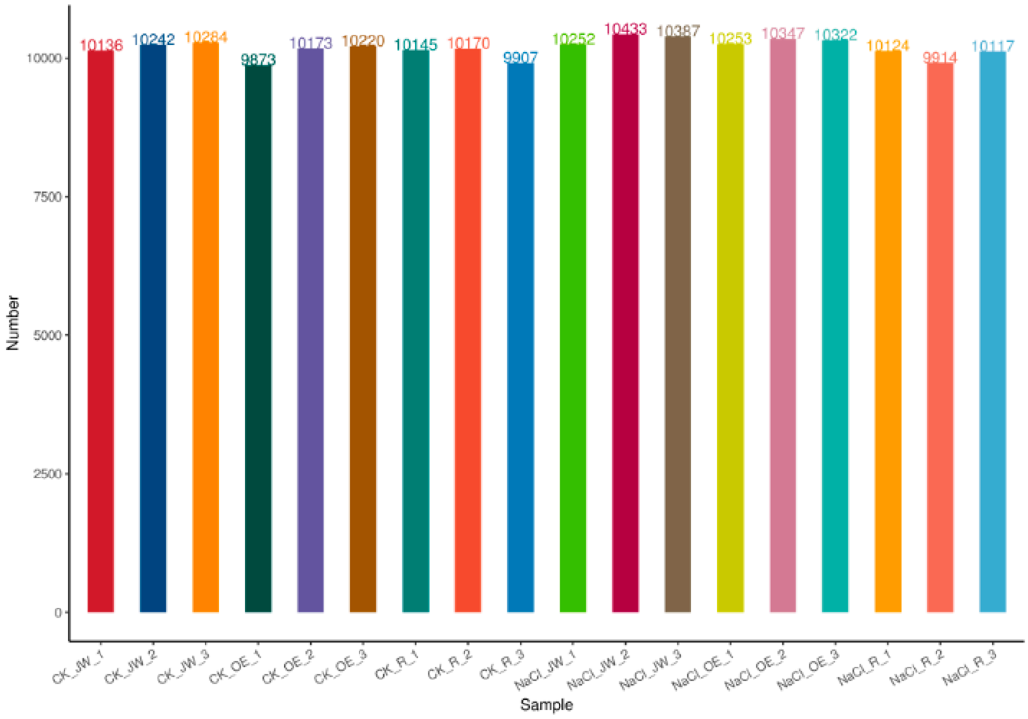

Figure S5. The number of proteins identified in different samples. The horizontal axis denotes the sample names, while the vertical axis indicates the corresponding protein quantities. CK_JW, JW wheat variety (WT) under normal condition. CK_OE, *TaNRX1-2D* overexpression transgenic wheat under normal condition. CK_R, *TaNRX1-2D* RNA interference transgenic wheat under normal condition. NaCl_JW, JW wheat variety (WT) under salt stress (200 mM NaCl) condition. NaCl_OE, *TaNRX1-2D* overexpression transgenic wheat under salt stress (200 mM NaCl) condition. NaCl_R, *TaNRX1-2D* RNA interference transgenic wheat under salt stress (200 mM NaCl) condition.

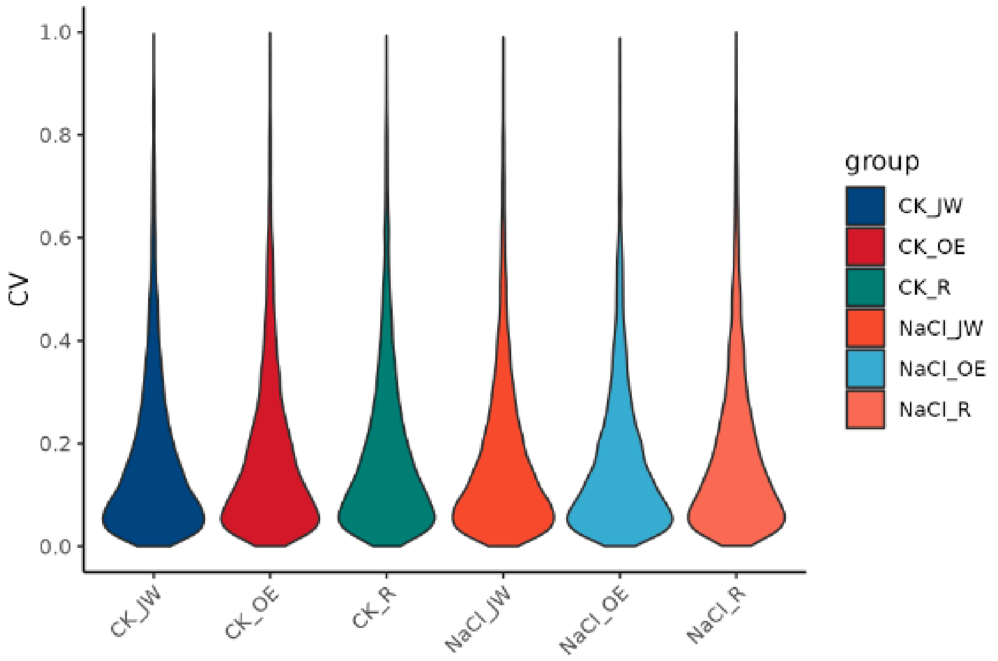

Figure S6. The violin plot of the coefficient of variation in protein quantity across different sample replicates. The horizontal axis denotes the sample names, while the vertical axis represents the corresponding coefficient of variation values for each sample group. CK_JW, JW wheat variety (WT) under normal condition. CK_OE, *TaNRX1-2D* overexpression transgenic wheat under normal condition. CK_R, *TaNRX1-2D* RNA interference transgenic wheat under normal condition. NaCl_JW, JW wheat variety (WT) under salt stress (200 mM NaCl) condition. NaCl_OE, *TaNRX1-2D* overexpression transgenic wheat under salt stress (200 mM NaCl) condition. NaCl_R, *TaNRX1-2D* RNA interference transgenic wheat under salt stress (200 mM NaCl) condition.
