# Supplementary material for: The Wheat Nucleoredoxin TaNRX1-2D Gene Ameliorates Salt Tolerance in Wheat (Triticum aestivum L.)
Source: Plants (Basel). 2026 Jan 4;15(1):146. doi: 10.3390/plants15010146 (PMC12787453; doi:10.3390/plants15010146)
Supplement: Supplementary file 1 [file plants-15-00146-s001.zip › Supplemental Table S1.pdf]

Table S1. Information of primers used in this study

Primer	Sequence(5'-3')	Usage
pABAi-(ABRE+ACGT)-F	gaaaagcttgaattc**gagctc**CCTCACGCGGTCACGTCC	Yeast one-hybrid
pABAi-(ABRE+ACGT)-R	agcacatgcctcgag**gtcgac**GGGACGTGACCGCGTGAG	
pGADT7-TaERD15L-3B-F	gccatggaggccagt**gaattc**ATGGAGGTGGTGACCGGC	
pGADT7-TaERD15L-3B-R	cagctcgagctcgat**ggatcc**TTAGCGAGGCTGCTGGATGG	
T7	TAATACGACTCACTATAGGG	
3-AD	AGATGGTGCACGATGCACAG	
0800-LUC-(ABRE+ACGT)-F	ctatagggcgaattg**ggtacc**CCTCACGCGGTCACGTCC	Dual-luciferase reporter assay
0800-LUC-(ABRE+ACGT)-R	atcgataccgtcgac**ctcgag**TTCTCTCCAAATGAAATGAACTTCC	
0800-LUC-ABRE-F	ctatagggcgaattg**ggtacc**CCTCACGCGGTCACCTCA	
0800-LUC-ABRE-R	atcgataccgtcgac**ctcgag**TTCTCTCCAAATGAAATGAACTTCC	
0800-LUC-ACGT-F	ctatagggcgaattg**ggtacc**GTCACGTCCCGTCACGTCC	
0800-LUC-ACGT-R	atcgataccgtcgac**ctcgag**TTCTCTCCAAATGAAATGAACTTCC	
62SK-TaERD15L-3B-F	cgctctagaactagt**ggatcc**ATGGAGGTGGTGACCGGC	
62SK-TaERD15L-3B-R	ggtaccgggcccccc**ctcgag**TTAGCGAGGCTGCTGGATGG	
pGBKT7-TaERD15L-3B-F	atggccatggaggcc**gaattc**ATGGAGGTGGTGACCGGC	Transcriptional activity
pGBKT7-TaERD15L-3B-R	atgcggccgctgcag**gtcgac**TTAGCGAGGCTGCTGGATGG	
pCambia1302-TaERD15L-3B-F	atctcgagctcaagc**ttcgaa**ATGGAGGTGGTGACCGGC	subcellular localization
pCambia1302-TaERD15L-3B-R	ccgtcgactgcagaa**ttcgaa**GCGAGGCTGCTGGATGGT	
VIGS-γ-TaERD15L-3B-F	TGTTGGATGTGATGATTCTTCTTCCGTTTCTAAGGAAGTTTAAAA[illegible]	BSMV-VIGS
VIGS-γ-TaERD15L-3B-R	ATCTGATCAAACATTTTTTTTTTTTTTAACCACCACCACCGTCTG	
TaCAT2-B-F	AGATCAGTGCAGTGAGAGTGAG	Cloning of genes
TaCAT2-B-R	ATCCATCATCCATCCACATCAC	
N-Luc-TaNRX1-D-F	acgggggacgagctc**ggtacc**ATGGCGGCGTCCTCCCCC	Luciferase complementation assays
N-Luc-TaNRX1-D-R	cgcgtacgagatctg**gtcgac**GGCCTTCCTGCAGACGTCTC	
C-Luc-TaCAT2-B-F	tacgcgtcccggggc**ggtacc**ATGGATCCCTGCAAGTTCCG	
C-Luc-TaCAT2-B-R	acgaaagctctgcag**gtcgac**TCACATGCTTGGCTTCACGTT	
nYFP-TaNRX1-D-F	cccaggcctactactagt**ggatcc**ATGGCGGCGTCCTCCCCCAC	bimolecular fluorescence complementation
nYFP-TaNRX1-D-R	agcggtaccctcgag**gtcgac**CTAGGCCTTCCTGCAGACG	
cYFP-TaCAT2-B-F	tggcgcgccactagt**ggatcc**ATGGATCCCTGCAAGTTCCG	
cYFP-TaCAT2-B-R	gaggtcgacagtact**atcgat**CATGCTTGGCTTCACGTTGA	
qPCR-TaERD15L-3B-F	CTGGGGGATCGAGAAGTGGT	RT-qPCR
qPCR-TaERD15L-3B-R	TTAGCGAGGCTGCTGGATG	
qPCR-TabHLH-6D-F	ATTCGCATGGAGGAGGCAAT	
qPCR-TabHLH-6D-R	CTCAAGAGGTCACTCGAGCC	
qPCR-TaNRX1-D-F	GTTGGCAGTTCCTTTCTCTGACTCC	
qPCR-TaNRX1-D-R	TCACTCACAAACTCAACTCCTTCG	
qPCR-TaActin-F	ACCTTCAGTTGCCCAGCAAT	
qPCR-TaActin-R	CAGAGTCGAGCACAATACCAGTTG	

Note: The sites of restriction are shown in bold.
