# Supplementary material for: The Wheat Nucleoredoxin TaNRX1-2D Gene Ameliorates Salt Tolerance in Wheat (Triticum aestivum L.)
Source: Plants (Basel). 2026 Jan 4;15(1):146. doi: 10.3390/plants15010146 (PMC12787453; doi:10.3390/plants15010146)
Supplement: Supplementary file 1 [file plants-15-00146-s001.zip › Supplemental Table S2.pdf]

Table S2. Information of candidate transcription factors

Number	Gene ID	Functional annotation
1	TraesCS6D02G038400	basic helix-loop-helix transcription factor
2	TraesCS7B02G148000	serine-glyoxylate aminotransferase
3	TraesCS4D02G135000	ribulose bisphosphate carboxylase/oxygenase activase
4	TraesCS6D02G378000	magnesium transporter MgtE-like
5	TraesCS3D02G403300	bidirectional sugar transporter SWEET1a-like
6	TraesCS6D02G234700	dehydrin COR410-like
7	TraesCS5B02G353200	chlorophyll a-b binding protein of LHCII type 1-like
8	TraesCS2A02G207900	cytochrome b6-f complex iron-sulfur subunit
9	TraesCS3B02G409300	protein Early responsive to dehydration 15-like
10	TraesCS3B02G471700	metacaspase-1-like
11	TraesCS1B02G311200	serine/threonine-protein kinase STN8
12	TraesCS2A02G324500	methyltransferase type 11
13	TraesCS4D02G006200	5-amino-6-(5-phospho-D-ribitylamino)uracil phosphatase
14	TraesCS5B02G162800	Ribulose bisphosphate carboxylase small chain
15	TraesCS6D02G367200	Bidirectional sugar transporter SWEET
16	TraesCS6D02G066700	Peptidyl-prolyl cis-trans isomerase
17	TraesCS6A02G221500	integral membrane metal-binding family protein
18	TraesCS1B02G179000	E3 ubiquitin-protein ligase RING1-like
19	TraesCS2B02G267500	oxygen-evolving enhancer protein 2
20	TraesCS7A02G126500	serine/threonine-protein phosphatase PP1
21	TraesCS6B02G344000	universal stress protein PHOS34-like
