# Supplementary material for: The Wheat Nucleoredoxin TaNRX1-2D Gene Ameliorates Salt Tolerance in Wheat (Triticum aestivum L.)
Source: Plants (Basel). 2026 Jan 4;15(1):146. doi: 10.3390/plants15010146 (PMC12787453; doi:10.3390/plants15010146)
Supplement: Supplementary file 1 [file plants-15-00146-s001.zip › Supplemental Table S3.pdf]

Table S3. Protein quality assessment results

Samples	Protein concentration（μg/μl）	Protein volume（μl）	Total quantity of protein（μg）	Detection result
CK-JW-1	6.94	95.00	658.88	A
CK-JW-2	6.92	95.00	657.05	A
CK-JW-3	7.07	95.00	671.48	A
CK-OE-1	8.24	95.00	782.84	A
CK-OE-2	8.42	95.00	800.03	A
CK-OE-3	8.25	95.00	783.88	A
CK-R-1	6.33	95.00	601.25	A
CK-R-2	6.34	95.00	601.94	A
CK-R-3	6.37	95.00	605.49	A
NaCl-JW-1	6.29	95.00	597.36	A
NaCl-JW-2	6.28	95.00	596.33	A
NaCl-JW-3	6.26	95.00	594.72	A
NaCl-OE-1	6.49	95.00	616.84	A
NaCl-OE-2	6.50	95.00	617.52	A
NaCl-OE-3	6.57	95.00	624.17	A
NaCl-R-1	7.70	95.00	731.06	A
NaCl-R-2	7.66	95.00	727.97	A
NaCl-R-3	7.64	95.00	726.25	A

Note: "A" indicates that the sample meets the required quality standards.
