# Supplementary material for: The Wheat Nucleoredoxin TaNRX1-2D Gene Ameliorates Salt Tolerance in Wheat (Triticum aestivum L.)
Source: Plants (Basel). 2026 Jan 4;15(1):146. doi: 10.3390/plants15010146 (PMC12787453; doi:10.3390/plants15010146)
Supplement: Supplementary file 1 [file plants-15-00146-s001.zip › Supplemental Table S4.pdf]

Table S4. Identification of differentially expressed proteins in leaves of *TaNRX1-2D* transgenic and WT wheat plants

Number	Protein ID	Functional annotation	CK_OE_1	CK_OE_2	CK_OE_3	NaCl_OE_1	NaCl_OE_2	NaCl_OE_3	CK_R_1	CK_R_2	CK_R_3	NaCl_R_1	NaCl_R_2	NaCl_R_3
1	TraesCS3D02G260300	26 kDa endochitinase 1-like	38905.8057	27240.49226	22518.63633	11899.41749	14029.74923	16717.57661	28825.19227	31274.4	25469.47135	58850.06644	74850.27812	74026.12548
2	TraesCS1A02G270400	uncharacterized protein	0	0	0	7377.803831	6931.62603	7882.363915	5158.727408	0	4995.764369	10790.3097	11998.24063	12679.1143
3	TraesCS1A02G122800	hexokinase-7-like	2519.541558	4741.122758	3823.281537	0	0	0	890.1452313	1460.54	0	0	3406.499289	4458.631845
4	TraesCS1B02G394500	non-specific lipid-transfer protein 2-like	0	0	0	7881.162432	13315.44847	12223.34141	6067.802939	8898.71	8476.216464	0	0	0
5	TraesCS2A02G110300	thaumatin-like protein	40917.40788	24622.37387	32912.34695	8747.209639	14267.95761	9240.823888	16642.9224	8180.81	17138.38261	49752.24782	45606.27223	51385.57334
6	TraesCS2A02G274700	probable aldo-keto reductase 2	4600.911423	9884.040791	0	16692.58959	24390.02971	18043.26812	0	5219.69	4160.685343	9472.138649	10989.20624	15035.71069
7	TraesCS2D02G199100	polyol transporter 5-like	22773.36048	22135.14549	23152.2259	7795.592649	5756.30609	5857.490824	9672.721676	11210.7	8759.346697	23368.73783	21458.98668	25606.10138
8	TraesCS3B02G415900	amidophosphoribosyltransferase	40007.52055	58427.18621	54637.31236	0	0	0	70924.95681	70721.7	97650.12302	31695.49059	37344.58207	42189.51292
9	TraesCS3D02G476500	glucan endo-1,3-beta-glucosidase GIII-like	66608.4296	59999.35025	57387.74362	21444.5142	10656.87857	12507.34187	29407.33911	17733.4	13576.69492	111529.3512	109484.7291	111461.5346
10	TraesCS4A02G099300	THO complex subunit 4A-like	0	16126.39262	10060.04467	30147.62653	41461.4502	29010.44954	0	8661.5	10800.20977	32269.86957	32462.14131	29619.9961
11	TraesCS4B02G063200	uncharacterized protein	0	0	0	2976.172148	3599.282155	4088.442445	0	0	0	4540.474014	3974.036912	3196.466011
12	TraesCS5A02G213200	transmembrane protein 87B-like	0	0	0	5925.206665	9105.085586	6280.650444	0	0	0	8573.538933	5598.063762	9300.824772
13	TraesCS5B02G223700	cinnamoyl-CoA reductase 1-like	0	0	0	16110.7322	16857.97367	12906.69415	0	0	0	11938.04271	8178.747798	10553.24415
14	TraesCS6A02G132300	L-type lectin-domain containing receptor kinase SIT2-like	13781.36837	8904.771457	9812.850016	3668.937061	6686.032431	2942.592971	13478.51416	11208.8	12754.33511	4542.154923	7034.060294	0
15	TraesCS6A02G395500	hypothetical protein CFC21_085370	4269.724435	2723.307339	1630.061776	0	0	0	0	0	0	3699.886165	5489.620109	4231.946565
16	TraesCS7B02G351900	non-specific lipid-transfer protein-like	8524.456527	9492.896891	9331.763983	17777.55916	23056.04115	20443.3337	30587.57515	28660.5	17726.97827	7778.427878	12697.27717	10548.73191
17	TraesCS3A02G517100	wheatwin-1 precursor	3859.6026	6005.181224	5071.063574	1636.807365	1454.498434	0	4992.976987	3726.05	1741.216228	17516.91889	20032.61251	18180.54656
18	TraesCS5A02G183300	pathogenesis-related protein 1-3	21133.41116	25646.81351	30477.13176	7970.921247	8004.98024	9263.253409	7364.438267	12031.4	4007.248274	127963.3136	127008.1481	139929.8046
19	TraesCS5D02G145300	non-specific lipid-transfer protein	31091.99043	23296.62222	31177.76394	74327.32625	84744.11864	88643.60395	69752.04022	63548.4	62595.70921	38297.36401	22870.30063	23910.098
20	TraesCS2B02G079300	ribulose-1,5-bisphosphate carboxylase/oxygenase small subunit	239777.1442	282525.3179	271206.8575	77130.86964	77068.25091	84318.231	206433.5206	189249	163886.0198	50310.74016	77765.89527	83227.09923

Note: CK_OE represents transgenic wheat with overexpression of *TaNRX1-2D* gene under normal conditions; NaCl_OE represents transgenic wheat with overexpression of *TaNRX1-2D* gene under salt stress treatment; CK_R represents transgenic wheat with RNA interference of *TaNRX1-2D* gene under normal conditions; NaCl_R represents transgenic wheat with RNA interference of *TaNRX1-2D* gene under salt stress treatment.
